# Intranasal fentanyl to reduce pain intensity associated with retinopathy of prematurity screening in preterm infants: study protocol for a randomized controlled trial

**DOI:** 10.1186/s12887-026-07328-x

**Published:** 2026-07-15

**Authors:** Helen McCord, Tim Disher, Véronique Promelle, Souvik Mitra, Balpreet Singh, Cameron Bruce, Carmen Peckham, Emily Neil, Marsha Campbell-Yeo

**Affiliations:** 1 School of Nursing, Faculty of Health Dalhousie, Dalhousie University, 5869 University Avenue, PO Box 15000, Halifax, NS Canada; 2 Neonatal Research, Research Services IWK Health, Sandpiper Analytics Halifax, 5850/5980 University Avenue Halifax, Halifax, NS B3K 6R8 Canada; 3 Pediatric Ophthalmology and Strabismus, Department of Ophthalmology and Visual Sciences, IWK Health and Dalhousie University, 5850/5980 University Avenue Halifax, Halifax, NS B3K 6R8 Canada; 4 Department of Pediatrics, BC Children’s Hospital, 4500 Oak Street, Vancouver, BC V6H 3N1 Canada; 5 Department of Pediatrics, IWK Health, 3rd Floor, 5850/5980 University Avenue, Halifax, NS B3K 6R8 Canada; 6 MOM-LINC Lab, IWK Health, 8th Floor Children’s Site 5850/5980 University Avenue, Halifax, NS B3K 6R8 Canada; 7 IWK Health Parent Partner, Neonatal Intensive Care Unit (NICU), IWK Health, 3rd Floor, 5850/5980 University Avenue, Halifax, NS B3K 6R8 Canada; 8 Neonatal Nurse Practitioner Dalhousie University, Halifax, NS Canada

**Keywords:** Preterm infant, Neonatal pain, Procedural pain, Retinopathy of prematurity, Intranasal fentanyl, Randomized controlled trial, Neonatal intensive care unit

## Abstract

**Background:**

Preterm infants are frequently exposed to painful procedures associated with adverse short- and long-term neurodevelopmental outcomes. Retinopathy of prematurity (ROP) screening is a necessary examination that is associated with moderate-to-high pain intensity scores and physiologic instability, despite the use of non-pharmacologic comfort measures and topical anesthesia. Current strategies have not reliably reduced procedural pain to mild or no pain levels, highlighting an important gap in neonatal pain management. Intranasal fentanyl provides rapid analgesia without requiring intravenous access and may offer a feasible option for procedural pain management in this population.

**Methods:**

This study is a single-center, randomized, double-blind, placebo-controlled trial conducted in a level III neonatal intensive care unit. Preterm infants born at ≤ 31 weeks’ gestational age and/or with birth weight < 1250 g who are scheduled for clinically indicated ROP screening will be randomly assigned (1:1) to receive intranasal fentanyl (2 µg/kg) or intranasal normal saline placebo, administered 10 min prior to the examination, in addition to topical anesthesia and standard comfort measures. The primary outcome is pain intensity measured using the Premature Infant Pain Profile–Revised (PIPP-R) during the first 30 s following speculum insertion. Secondary outcomes include ongoing pain response, pain recovery, cry duration, physiologic recovery, salivary cortisol response, exam duration, adequacy of retinal visualization, and adverse events. Outcomes will be assessed using synchronized video, audio, and physiologic monitoring, with analyses conducted under an intention-to-treat framework.

**Discussion:**

This trial will provide high-quality evidence regarding the effectiveness and safety of intranasal fentanyl for procedural pain relief during ROP screening in preterm infants. Findings may inform future clinical guidelines and contribute to improved pain management practices for this population.

**Trial registration:**

ClinicalTrials.gov, NCT07112430. https://clinicaltrials.gov/study/NCT07112430 Prospectively registered on August 8, 2025.

**Supplementary Information:**

The online version contains supplementary material available at https://doi.org/10.1186/s12887-026-07328-x.

## Administration information

**Table:** 

Title {1a}	Intranasal Fentanyl to Reduce Pain Intensity Associated with Retinopathy of Prematurity Screening in Preterm Infants: study protocol for a randomized controlled trial
**Trial registration** {4}	**ClinicalTrials.gov**: NCT07112430 **Date of registration**: 08 August 2025
**Protocol version** {2}	May 15, 2026
**Funding** {7a}	This is an investigator-initiated study supported by internal academic research funds associated with the Principal Investigator’s doctoral research program at Dalhousie University. No industry funding has been received. Study medications (fentanyl and normal saline placebo) are supplied through routine hospital pharmacy services at IWK Health. **Role of the funder**: The funder has no role in study design; collection, management, analysis, or interpretation of data; writing of the report; or the decision to submit the report for publication.
**Author details** {3a}	**Helen McCord** School of Nursing, Faculty of Health Dalhousie Dalhousie University 5869 University Avenue PO Box 15,000 Halifax, Nova Scotia, Canada **Marsha Campbell-Yeo** School of Nursing, Faculty of Health Dalhousie Dalhousie University 5869 University Avenue PO Box 15,000 Halifax, Nova Scotia, Canada **Balpreet Singh** Department of Pediatrics, IWK Health 3rd Floor, 5850/5980 University Avenue Halifax, NS, B3K 6R8, Canada Email: balpreet.singh@iwk.nshealth.ca **Véronique Promelle** Pediatric Ophthalmology and Strabismus Department of Ophthalmology and Visual Sciences, IWK Health and Dalhousie University 5850/5980 University Avenue Halifax, NS, B3K 6R8, Canada Email: veronique.promelle@dal.ca **Souvik Mitra** Department of Pediatrics, BC Children’s Hospital 4500 Oak Street Vancouver, BC, V6H 3N1, Canada Email: souvik.mitra@cw.bc.ca **Tim Disher** Neonatal Research, Research Services IWK Health, 5850/5980 University Avenue Halifax, NS, B3K 6R8, Canada Sandpiper Analytics Halifax, Nova Scotia, Canada Email: tim.disher@dal.ca **Cameron Bruce** Research Coordinator, MOM-LINC Lab, IWK Health 8th Floor Children’s Site 5850/5980 University Avenue Halifax, NS, B3K 6R8, Canada Email: cameronj.bruce@iwk.nshealth.ca **Carmen Peckham** IWK Health Parent Partner, Neonatal Intensive Care Unit (NICU), IWK Health, Halifax, Nova Scotia, Canada Email: cghpeckham@gmail.com **Emily Neil** IWK Health Parent Partner, Neonatal Intensive Care Unit (NICU), IWK Health, Halifax, Nova Scotia, Canada Email: eventsemily@gmail.com
**Name and contact information for the trial sponsor**{3b}	Investigator-sponsored trial **Marsha Campbell-Yeo**, NNP-BC, PhD, FAAN School of Nursing, Faculty of Health Dalhousie Dalhousie University 5869 University Avenue PO Box 15,000 Halifax, Nova Scotia, Canada MOM-LINC Lab, IWK Health (Children’s Site) 5850/5980 University Avenue Halifax, NS, B3K 6R8, Canada Email: marsha.campbell-yeo@dal.ca
**Role of sponsor** {3c}	The sponsor (Dr. Marsha Campbell-Yeo) provides oversight of study conduct and ensures regulatory and ethical compliance. The Principal Investigator (Helen McCord), as a doctoral trainee, is responsible for day-to-day trial coordination, data collection, and protocol implementation under the supervision of the sponsor. The sponsor and Principal Investigator are responsible for study design; data management, analysis, and interpretation; manuscript preparation; and the decision to submit results for publication. No commercial entity has any role in study conduct or reporting.

## Background and rationale {9a}

Painful procedures are a common and unavoidable part of neonatal intensive care unit (NICU) care, especially in preterm infants who often require repeated and invasive interventions throughout hospitalization. Repeated exposure to procedural pain during this critical developmental period has been associated with adverse short- and long-term outcomes, including altered brain development, dysregulated stress responses, and later neurodevelopmental and behavioral impairments [1–6]. Although awareness of neonatal pain and implementation of pain management strategies have improved substantially over time, evidence suggests that currently available non-pharmacologic interventions alone are often insufficient to provide adequate analgesia during more invasive procedures [7–9].

Retinopathy of prematurity (ROP) screening is one such procedure, routinely performed in preterm infants and often repeated multiple times throughout an infant’s NICU stay. The examination involves eyelid speculum insertion and scleral depression to visualize the retina. Evidence indicates that this examination is associated with higher pain intensity and distress, reflected in elevated pain scores, physiologic instability, prolonged crying time [10–13] and neurophysiologic evidence of noxious-evoked pain activity [14,15]. Current standard care commonly includes oral sucrose, non-nutritive sucking, swaddling, facilitated tucking, and topical ophthalmic anesthetic drops; however, these approaches have not reliably reduced pain to mild or no pain levels during ROP examinations [16,17]. Other supportive interventions, such as skin-to-skin care, may provide analgesic benefit during some neonatal procedures but are difficult to implement during ROP screening because of positioning requirements and the need for direct ophthalmologic access. As a result, approaches to pain management during ROP examinations remain variable across NICUs, and no clear consensus currently exists regarding optimal analgesic management for this procedure.

Intranasal (IN) fentanyl has emerged as a promising agent for procedural pain relief in pediatric populations due to its rapid onset, ease of administration, and avoidance of intravenous access [18–20]. Intravenous opioids, including fentanyl, have demonstrated analgesic efficacy for procedural and postoperative pain in neonates however, their use during brief bedside procedures such as ROP screening is limited by practical and safety considerations. Establishing intravenous access may itself cause pain and distress, may not be readily available at the time of examination, and carries risks related to cardiorespiratory depression in preterm infants. In addition, the high volume of infants requiring ROP screening relative to limited physician time underscores the need for analgesic strategies that are efficient and feasible to implement without adding significant procedural burden or resource demands. In contrast, intranasal administration offers a non-invasive route with rapid systemic absorption and avoids the need for venous access.

While intranasal fentanyl has been extensively studied and adopted in older children and emergency settings [18–23], there is limited evidence evaluating its safety and effectiveness in infants. Existing trials of intranasal fentanyl during ROP screening report inconsistent findings, underscoring the need for further high-quality evidence in this population [13, 24]. A recent systematic review identified two randomized controlled trials evaluating intranasal fentanyl for procedural pain in infants, both conducted during ROP screening [25, 26]. The review concluded that the certainty of evidence is low, findings are inconsistent, and additional well-designed randomized controlled trials with standardized outcome assessment and robust safety monitoring are required. Given the potential for opioid-related adverse effects in preterm infants, including respiratory depression or apnea, bradycardia, hypotension, and rare chest wall rigidity, existing neonatal evidence remains limited and highlights the importance of careful safety evaluation.

In contrast, intranasal saline placebo is not expected to provide analgesic benefit or medication-related adverse effects; however, infants in this group may still experience pain associated with routine ROP screening performed under standard care conditions.

Given the ongoing variability in neonatal pain management practices, the limited and inconsistent evidence base, and the need for effective and feasible analgesia during ROP screening, a rigorously designed randomized controlled trial is warranted. This study aims to evaluate the effectiveness and safety of intranasal fentanyl administered prior to ROP screening in preterm infants using standardized pain assessment, physiologic monitoring, and comprehensive safety evaluation. Findings from this trial may inform future clinical guidelines and contribute to improved evidence-based procedural pain management practices for this vulnerable population.

### Objectives

The primary objective is to examine the efficacy of intranasal fentanyl plus standard care compared to intranasal saline plus standard care in reducing immediate pain intensity, as measured by the PIPP-R [23].

Secondary objectives are to determine the efficacy of intranasal fentanyl plus standard care compared to intranasal saline plus standard care on (a) ongoing pain response throughout the procedure measured using the PIPP-R [23]; (b) cry duration; (c) stress response (salivary cortisol); (d)incidence of adverse events; (e) the proportion of infants experiencing no to low pain during and following the ROP exam; (f) the duration of the ROP exam; and (g) the time to physiologic recovery (return to baseline heart rate and oxygen saturation).

#### Hypotheses

##### Primary

Infants randomized to intranasal fentanyl plus standard care, compared with intranasal saline plus standard care, will demonstrate significantly lower pain intensity during retinopathy of prematurity (ROP) screening, as measured by PIPP-R scores during the first 30 s following speculum insertion.

##### Secondary

Infants receiving intranasal fentanyl plus standard care, compared with those receiving intranasal saline plus standard care, will demonstrate (a) lower ongoing pain and faster pain recovery as reflected by reduced PIPP-R scores during the procedure and at 1 and 5 min post-examination; (b) shorter time to physiologic recovery and reduced cry duration during and following the procedure; (c) a lower salivary cortisol response; and (d) a greater proportion of infants experiencing low-to-mild pain (PIPP-*R* ≤ 6). In addition, intranasal fentanyl will not prolong examination duration, impair retinal visualization or completion of the examination, nor result in a higher incidence of adverse events (including apnea, bradycardia, hypotension, chest-wall rigidity, or need for airway support).

## Methods

### Trial design

A single-centre, randomized, double-blind, placebo-controlled, parallel-group superiority trial design will be utilized. Preterm infants undergoing clinically indicated ROP screening will be randomized in a 1:1 ratio to receive either (i) intranasal fentanyl or (ii) intranasal normal saline (placebo), in addition to standard comfort measures. The study protocol has received institutional Research Ethics Board approval and authorization from Health Canada.

### Study setting

**Fig. 1:**
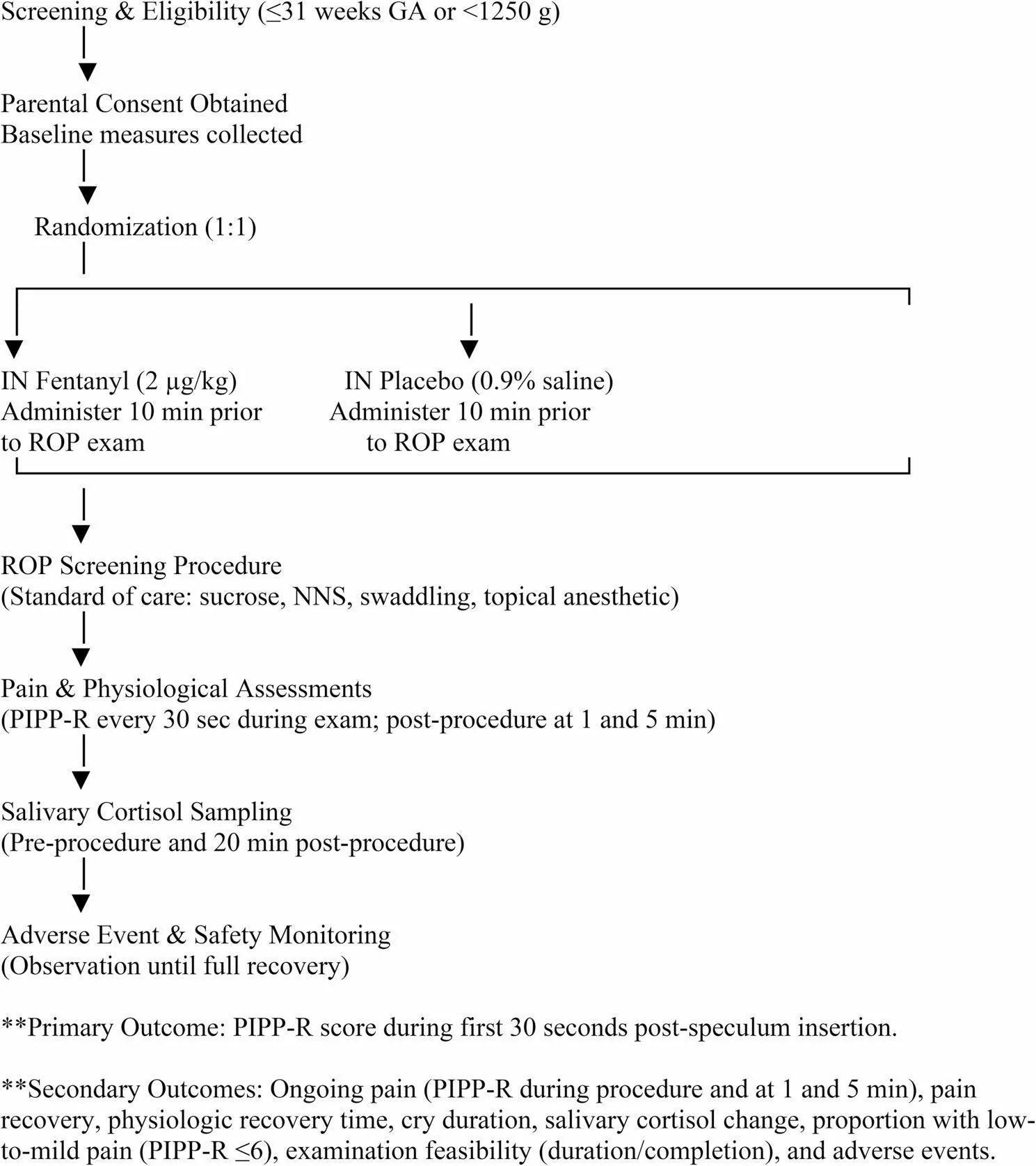
Study design schematic. Single-centre, randomized (1:1), double-blind, placebo-controlled trial of intranasal fentanyl (2 micrograms/kg) versus saline administered 10 min prior to ROP screening

The study will be conducted in the neonatal intensive care unit (NICU) at IWK Health in Halifax, Nova Scotia, Canada. This is a 40-bed, tertiary-level referral unit providing care to preterm and medically complex infants (Fig. 1).

### Eligibility criteria

Infants will be eligible for participation if they are born at ≤ 31 weeks’ gestational age and/or with a birth weight ≤ 1250 g and are scheduled to undergo ROP screening according to current national (Canadian) ROP screening guidelines and routine NICU care. Infants must be clinically stable at the time of screening. Infants may be enrolled for a single study visit during one scheduled ROP examination. Eligibility does not depend on whether the examination is the infant’s first or a subsequent ROP screening.

Clinical stability will be determined in consultation with the attending neonatal team and is defined as no requirement for acute resuscitative support (bag-mask ventilation, intubation, or chest compressions) within the preceding 24 h, with maintenance of stable cardiorespiratory parameters on baseline respiratory support. Written informed consent must be obtained from a parent or legally authorized representative prior to enrollment.

Infants will be excluded if they have congenital malformations affecting the nasal passages that would interfere with intranasal administration, have received systemic opioids, benzodiazepines, barbiturates, or other sedative or analgesic medications within 24 h prior to the scheduled ROP examination, or have a known previous adverse reaction to fentanyl.

### Concomitant care

All infants will continue to receive routine neonatal intensive care as clinically indicated throughout the study period. Standard of care measures for ROP screening, including topical ophthalmic anesthetic drops and non-pharmacologic comfort measures (oral sucrose, non-nutritive sucking, swaddling) will be provided to all participants. The use of additional systemic analgesics, opioids, sedatives, or anesthetic agents for the purpose of pain control during the ROP examination is prohibited. Medications or interventions required for clinical emergencies or for the infant’s medical management are permitted at all times and will be documented; such events will only be classified as protocol deviations if they result in non-adherence to study procedures.

### Recruitment

Eligible infants will be identified prospectively using the IWK Health NICU ROP screening schedule and daily clinical review to minimize missed opportunities for enrollment. Consent for the research team to approach potentially eligible participants will be facilitated by the clinical team. Written informed consent will be obtained prior to enrollment by trained members of the research team who are not part of the infant’s direct clinical care, from the parent(s) or other legally authorized representative(s).

### Randomization and blinding

Participants will be randomized in a 1:1 ratio to receive either intranasal fentanyl or intranasal placebo using a computer-generated randomization sequence prepared and maintained by the IWK Health Pharmacy. Variable permuted block sizes of 4 and 6 will be used, with stratification by gestational age at birth using predefined strata (< 28⁰⁄₇ weeks and 28⁰⁄₇–30⁶⁄₇ weeks). These strata are included as prognostic factors to improve precision of treatment effect estimation; analyses are not powered within strata.

Allocation concealment will be ensured through centralized, pharmacy-controlled randomization. Pharmacy staff will assign the next sequential study subject identifier within the appropriate gestational age stratum and prepare and dispense study medication in identical syringes labeled only with the study subject identifier and required dispensing information, with no indication of treatment assignment. The randomization sequence will not be accessible to investigators or clinical staff involved in participant enrollment, intervention administration, outcome assessment, or data analysis.

Blinding will be maintained by ensuring that intranasal fentanyl and 0.9% saline placebo are identical in appearance, volume, labeling, and method of administration, and are delivered using the same mucosal atomization device. After assignment, investigators, clinical care providers, outcome assessors, families/participants, and data analysts will remain blinded to group allocation until completion of data analysis, unless unblinding is required for participant safety. Emergency unblinding will occur only when knowledge of treatment allocation is deemed necessary for clinical management by the responsible clinical physician in consultation with the study team and will be conducted through the IWK Health Pharmacy. The reason for unblinding, the individual requesting unblinding, and the timing of unblinding will be documented. The extent of unblinding (e.g., clinical team only versus outcome assessors or study investigators) will also be recorded to preserve trial integrity and will be reported to the Data Safety Monitoring Board (DSMB) and the Research Ethics Board (REB).

### Interventions

All infants will receive standard of care measures for ROP screening, including topical ophthalmic anesthetic drops (tetracaine 0.5%) and non-pharmacologic comfort measures routinely used during ROP screening: oral sucrose, non-nutritive sucking, swaddling. Pupil dilation one hour prior to ROP examination will be performed according to standard of care and is not part of the research intervention.

#### Intervention group

Infants randomized to the intervention group will receive intranasal fentanyl at a dose of 2 mcg/kg, administered via a mucosal atomization device into a single nostril 10 min prior to the ROP examination.

#### Control group

Infants randomized to the control group will receive intranasal 0.9% normal saline at a volume of 0.2 mL/kg, administered using the same device and timing as the intervention group.

Intranasal saline was selected as the comparator to permit double blinding and to isolate the analgesic effect of intranasal fentanyl beyond standard of care measures, which are provided to all infants as part of routine care. No separate intervention manual is required. Detailed administration procedures are described in this protocol and are implemented according to standard institutional medication administration policies. Study materials, including case report forms and analytic code, will be available from the corresponding author upon reasonable request.

### Site and provider eligibility

This is a single-centre trial conducted exclusively at IWK Health; therefore, no additional site eligibility criteria apply. Study medication will be prepared by the hospital pharmacy and administered by trained neonatal clinical staff (e.g., neonatal nurses, nurse practitioners, or physicians) in accordance with institutional policies for medication administration. No specialized certification beyond standard clinical competencies is required to deliver the intervention (Table 1).

**Table 1:** SPIRIT schedule of enrolment interventions, and assessments

	TRIAL PERIOD							
	Enrollment		Post-randomization					Close-out
TIMEPOINT^b^	-t_i_ to 0 (Screening and consent)	0 (Randomization)	t_1_ -20 min (Cortisol)	t_2_ −10 min (Study drug)	t_3_ 0 (Speculum insertion)	t_4_ + 1 & +5 min (Recovery)	t_5_ + 20 min (Cortisol)	t_x_ 0–4 h (Safety)
ENROLLMENT:								
Eligibility screen	X							
Informed consent	X							
Baseline clinical data collection	X							
Randomization		X						
INTERVENTION/ COMPARATOR:								
Intranasal fentanyl (2mcg/kg)^*c*^				X				X
Intranasal saline placebo^*d*^				X				
Standard care (sucrose, NNS, swaddling & topical anesthetic)					X			
ROP screening examination					X			
ASSESSMENTS:								
Salivary cortisol			X				X	
PIPP-R (primary outcome: first 30 s)					X			
PIPP-R (every 30 s during exam)					X			
PIPP-R (recovery at + 1 and + 5 min)						X		
Cry duration					X	X		
Physiologic monitoring (HR, SpO₂, RR, BP)					continuous	continuous		
Examination duration					X			
Adequacy of retinal visualization								X
Adverse events					X	X		X

Continuous physiologic monitoring is performed as part of routine NICU care. Adverse events are monitored during the procedure and for four hours following study drug administration

^a^ Recommended content can be displayed using various schematic formats

^b^ Target timepoints: −t₁ to 0 = screening/consent; 0 = randomization; t₁ = 20 min before procedure; t₂ = 10 min before procedure; t₃ = eyelid speculum insertion (start of ROP exam); t₄ = 1 and 5 min post-procedure; etc. = 20 min post-procedure; tₓ = 0–4 h post-drug administration

^c^ Single intranasal fentanyl dose administered once at t₂

^d^ Single intranasal saline placebo administered once at t₂

### Outcomes

#### Primary outcome

The primary outcome is pain intensity measured using the Premature Infant Pain Profile–Revised (PIPP-R). The measurement variable is the PIPP-R total score. The primary analysis will evaluate the between-group difference in mean PIPP-R score during the first 30 s following eyelid speculum insertion, defined as the moment the speculum is fully positioned and the ROP examination begins.

#### Secondary outcomes

Secondary outcomes include:

1. Ongoing pain response
  *Measurement variable:* PIPP-R total score
  *Analysis metric:* Mean score over repeated 30-second intervals during the examination
  *Aggregation:* Group means (with standard deviations)
  *Time points:* Every 30 seconds during the ROP examination
2. Pain recovery
  *Measurement variable:* PIPP-R total score
  *Analysis metric:* Mean score at each recovery time point
  *Aggregation:* Group means
  *Time points:* 1 minute and 5 minutes following speculum removal
3. Cry duration
  *Measurement variable:* Total seconds of audible crying
  *Analysis metric:* Mean duration per group
  *Aggregation:* Group means or medians (as appropriate)
  *Time points:* During the procedure and recovery period
4. Time to physiologic recovery (defined as return of heart rate and oxygen saturation to baseline)
  *Measurement variables:* Heart rate and oxygen saturation
  *Analysis metric:* Time to return to baseline values
  *Aggregation:* Median time to recovery
  *Time points:* Continuously recorded during and after the examination
5. Stress response (Salivary cortisol response measured before and after the procedure)
  *Measurement variable:* Salivary cortisol concentration
  *Analysis metric:* Change from pre- to post-procedure levels
  *Aggregation:* Mean change per group
  *Time points:* 20 minutes before and 20 minutes after the examination
6. Duration of the ROP examination
  *Measurement variable:* Total examination time (minutes)
  *Analysis metric:* Mean difference between groups
  *Aggregation:* Group means
  *Time points:* During the procedure
7. Adequacy of retinal visualization
  *Measurement variable:* Clinician-rated adequacy of examination
  *Analysis metric:* Proportion of adequate examinations
  *Aggregation:* Percentage per group
  *Time points:* At completion of each examination
8. Incidence of adverse events
  *Measurement variables:* Occurrence of apnea, bradycardia, hypotension, chest wall rigidity, or need for airway support
  *Analysis metric:* Incidence by group
  *Aggregation:* Proportions
  *Time points:* During the examination and within four hours post-administration

### Data collection methods

Synchronized video, audio, and physiologic data will be captured during the ROP examination using a research-grade monitoring system. Pain assessments will be conducted by trained assessors blinded to group allocation using video recordings labelled only with study identifiers. Pain will be measured using the Premature Infant Pain Profile–Revised (PIPP-R), a validated neonatal pain assessment tool that incorporates behavioral and physiologic indicators and has demonstrated good reliability and validity in preterm infants.

Baseline demographic and clinical data will be abstracted from the medical record, including gestational age, sex, birth weight, postmenstrual age, level of respiratory support, comorbidities, and cumulative exposure to prior painful procedures. Cumulative exposure to prior painful procedures will be recorded using predefined categories of tissue-breaking or potentially noxious interventions (e.g., heel lance, venipuncture, peripheral or central line insertion, endotracheal intubation), as detailed in Fig. Appendix. Salivary cortisol samples will be collected 20 min before and 20 min after the procedure using validated infant saliva collection swabs and processed according to standard laboratory procedures.

To promote data quality, all assessors will receive standardized training prior to study initiation. A subset of video recordings will be independently scored by a second blinded assessor to assess inter-rater reliability, and discrepancies will be reviewed by the study team. Data will be recorded using standardized case report forms and entered into a secure study database with range and consistency checks. Data collection forms, including case report forms and scoring templates, are available from the corresponding author upon reasonable request.

### Data management, security, and storage

All study data (video recordings, physiologic signals, and salivary cortisol results) will be de-identified and labeled using unique study identification numbers prior to analysis. Synchronized video, audio, and physiologic data will be captured using the EGI research-grade system during the ROP examination to support blinded PIPP-R scoring and cry-duration analyses, and stored on secure, password-protected institutional servers at IWK Health with access restricted to authorized study personnel. Salivary cortisol samples will be labeled with study IDs only and processed according to standard laboratory procedures, with resulting laboratory data entered into the study database. Clinical and outcome data will be entered into REDCap (Research Electronic Data Capture) using predefined variable definitions and data dictionaries, with built-in range, logic, and consistency checks to identify missing or implausible values. Key outcome variables will undergo secondary verification against source data (video files, physiologic recordings, and laboratory reports), and periodic data audits will be conducted by the study team and the sponsor’s research office. The master participant log linking study IDs to personal identifiers and authorization forms will be stored separately in a locked cabinet within a restricted-access office; any electronic linkage files will be maintained in encrypted, password-protected folders.

All electronic data will be stored on encrypted institutional servers with regular backups in accordance with IWK Health and Dalhousie University data security policies. Study records, including de-identified datasets, will be retained for a minimum of 25 years (or at least 15 years as required by Health Canada, whichever is longer) and then securely destroyed or permanently de-identified in accordance with institutional policy. Following publication, the de-identified dataset, data dictionary, and statistical code will be made available from the corresponding author upon reasonable request, subject to Research Ethics Board approval and appropriate data-sharing agreements.

### Participant retention and follow-up

Because this is a single-visit procedural study, the risk of loss to follow-up is minimal. Study procedures coincide with clinically indicated ROP screening, requiring no additional visits and minimal additional burden for families. Research staff will coordinate closely with clinical teams to ensure that study procedures are completed at the scheduled examination time. If a parent or legally authorized representative withdraws consent for the infant’s participation, if the infant is unable to receive the allocated intervention, or if deviations from the intervention protocol occur (e.g., incomplete drug administration, early termination of the examination), outcome data collected up to the time of discontinuation will be retained, unless the parent or legally authorized representative requests otherwise.

Incomplete drug administration will be defined as failure to deliver the full intended intranasal dose due to technical or clinical factors (e.g., device malfunction, loss of medication prior to delivery, or inability to complete administration). Minor medication loss during normal administration (e.g., sneezing or small-volume reflux) will not be considered incomplete dosing unless judged by the administering clinician to result in substantial dose loss.

The following data will be collected whenever feasible, regardless of protocol deviations: pain scores derived from available video recordings (PIPP-R), physiologic measures (heart rate, oxygen saturation), cry duration, examination duration, and adverse events. Salivary cortisol samples will be analyzed only if both pre- and post-procedure samples are obtained according to protocol. Reasons for withdrawal, protocol deviations, and missing data will be documented, and patterns of missingness will be evaluated in the statistical analysis.

### Sample size

The sample size for this study was determined using variability estimates from previous randomized controlled trials evaluating intranasal fentanyl in infants. One study reported standard deviations (SD) ranging from 2.1 to 2.3 across treatment groups, while another reported higher variability, with SDs of 2.6 in the control group and 3.5 in the fentanyl group [13, 24]. Neonatal pain studies more broadly often report SDs in the range of 1.7–2.5 for brief procedures such as heel lance and ROP examinations. Based on these findings, we selected a conservative SD of 2.0 for planning. A 2-point reduction in the Premature Infant Pain Profile–Revised (PIPP-R) score is considered clinically meaningful in this context. Using a two-sided α = 0.05, 90% power, equal allocation (1:1), and a two-sample comparison of means for the primary analysis, a total sample size of 46 infants (23 per group) is required to detect this difference assuming an SD of 2.0 (calculated using G*Power). To account for potential attrition due to withdrawal, missing salivary samples, unusable video recordings, or protocol deviations, the required sample size will be inflated by approximately 20–25%, resulting in a final target sample size of 58 infants (29 per group).

Recruitment feasibility was assessed using historical admission data from the IWK Health NICU, where a mean of approximately 62 infants born at ≤ 31 weeks’ gestation were admitted annually between 2022 and 2024. Based on recruitment rates observed in prior single-procedure neonatal medication trials at this site, an anticipated parental or legally authorized representative consent rate of 70–75% is expected, yielding approximately 43–47 enrollments per year. At this rate, the target sample size of 58 infants is achievable within 12–18 months. This timeline is supported by the unit’s established ROP screening program, the research team’s prior trial experience, and the minimal participant burden associated with a single, short-acting intervention integrated into routine care.

### Statistical analysis

All analyses will be conducted using appropriate statistical software. Descriptive statistics will be used to summarize baseline characteristics by treatment group. Continuous variables will be reported using mean, standard deviations (SD), median, interquartile ranges (IQR) and range. Categorical variables will be summarized as counts and percentages. Consistent with randomized trial reporting guidelines, no formal statistical testing will be performed to compare baseline characteristics between groups. All randomized participants will be included in the primary analysis according to the intention-to-treat principle and analyzed in the group to which they were allocated, regardless of whether the study drug was fully administered, the procedure was completed as planned, or other protocol deviations occurred. Participants with missing outcome data will remain in the analysis dataset, with missingness handled as described in the Missing data section.

### Primary outcome

The primary outcome, pain intensity measured using the Premature Infant Pain Profile–Revised (PIPP-R) during the first 30 s following speculum insertion, will be analyzed using a linear regression model with treatment group as the primary predictor. The treatment effect will be reported as the adjusted mean difference in PIPP-R score with 95% confidence intervals. The model will include prespecified covariates selected a priori: gestational age stratification group (< 28⁰⁄₇ weeks vs. 28⁰⁄₇–30⁶⁄₇ weeks), sex (binary), examination number (first vs. subsequent), and cumulative prior exposure to painful procedures. Gestational age will be modeled using the same categorical strata applied in randomization. Continuous covariates will be included as linear terms without data-driven transformation. Inference will be based on heteroskedasticity-robust standard errors, and the primary analysis model will not be modified based on distributional testing.

### Secondary outcomes

Secondary continuous outcomes (e.g., ongoing and recovery PIPP-R scores, cry duration, time to physiologic recovery, salivary cortisol change, and examination duration) will be analyzed using linear regression or non-parametric methods as appropriate. Repeated pain measures over time will be analyzed using linear mixed-effects models to account for within-infant correlation. Binary outcomes (e.g., adequacy of retinal visualization and occurrence of adverse events) will be analyzed using logistic regression, with results reported as odds ratios and 95% confidence intervals.

#### Harms and safety outcomes

Adverse events and serious adverse events will be summarized by treatment group using counts and proportions. Between-group comparisons will be conducted using chi-square or Fisher’s exact tests, as appropriate. Safety outcomes will be summarized descriptively and compared between groups using prespecified categorical or continuous methods consistent with the outcome type (e.g., chi-square or Fisher’s exact test for categorical variables and linear regression models for continuous measures).

#### Missing data

Patterns and extent of missing data will be summarized for all outcomes and covariates. The primary analysis will follow the intention-to-treat principle. Missing outcome or covariate data will be addressed using multiple imputation by chained equations (MICE) under a missing-at-random assumption. The imputation model will include treatment group, gestational age stratification group, sex, examination number, cumulative prior exposure to painful procedures, and available outcome measures. Imputed datasets will be analyzed using the prespecified linear regression model, with estimates combined using Rubin’s rules. Sensitivity analyses will include complete-case analysis to assess robustness of findings to assumptions about missing data. All statistical tests will be two-sided with a significance level of α = 0.05.

### Additional and exploratory analyses

Sensitivity analyses will be performed to assess the robustness of the primary findings. These will include analyses using alternative regression specifications with robust standard errors and complete-case analyses under different assumptions about missing data, as described in the Missing Data section. Exploratory subgroup analyses may be conducted to examine whether treatment effects differ by gestational age category at birth (< 28 weeks vs. 28–30⁶⁄₇ weeks), age at examination (before vs. after 38 weeks) or by exam number (first vs. subsequent ROP examination). These analyses will be considered hypothesis-generating and interpreted cautiously.

### Harms and safety monitoring.

All infants will undergo continuous cardiorespiratory monitoring as part of routine NICU care. Adverse events (AEs) and serious adverse events (SAEs) will be actively monitored during the procedure and for four hours following study drug administration and documented in case report forms. An independent Data Safety Monitoring Board (DSMB) will oversee participant safety and trial conduct. The DSMB will be composed of at least three members with expertise in neonatology, pediatric pharmacology or clinical trials, and biostatistics. All members will be independent of the study investigators, sponsor, and funders, and will have no financial or other competing interests related to the intervention under study. The DSMB will review accumulating safety data at prespecified intervals and on an ad hoc basis in the event of serious adverse events. Its primary responsibilities include monitoring rates and severity of adverse events, evaluating any emerging safety concerns, and making recommendations regarding trial continuation, modification, or termination based on risk–benefit considerations. Written recommendations will be provided to the Principal Investigator, who will communicate these to the Research Ethics Board and relevant regulatory authorities as required. A formal DSMB charter outlining membership, roles, review schedule, stopping considerations, and reporting procedures will be maintained by the study team and is available from the corresponding author upon reasonable request.

Harms will be defined and assessed using standard AE and SAE criteria. AEs will include any unfavorable medical occurrence temporally associated with study drug administration, whether considered related to the intervention. Prespecified events of interest include apnea, bradycardia, hypotension, chest wall rigidity, oxygen desaturation, and the need for airway or ventilatory support. SAEs will include any event that results in death, is life-threatening, requires escalation of care, or results in prolonged hospitalization. All AEs and SAEs will be recorded, assessed for severity and relatedness to the study intervention, and reported to the DSMB and REB in accordance with institutional policies.

Because the intervention consists of a single, weight-based dose administered prior to the procedure, dose modification is not planned. Administration of the study drug will be discontinued immediately if an infant experiences a serious adverse event or any clinical deterioration that, in the judgment of the attending clinician, warrants cessation of the study intervention (e.g., apnea requiring stimulation or ventilation, significant bradycardia, hypotension, or need for airway support). Parents or legally authorized representatives may withdraw their infant from the study at any time without impact on clinical care. Infants who discontinue the intervention will continue to receive standard comfort measures and will be followed for safety outcomes as appropriate.

### Interim analyses and stopping guidelines

No formal interim analyses for efficacy are planned due to the relatively small sample size, short duration of participant involvement, and the single-procedure nature of the intervention. Safety data will be reviewed periodically by the Data Safety Monitoring Board (DSMB). The DSMB may recommend early trial termination or protocol modification in the event of unexpected safety concerns, higher-than-anticipated adverse event rates, or feasibility issues that compromise study conduct. Any decision to stop the trial early will prioritize participant safety and will be made in consultation with the DSMB, Research Ethics Board, and study investigators.

### Adherence to intervention protocol

Adherence to the intervention protocol will be promoted through standardized pharmacy preparation of study medication, weight-based dosing, identical administration devices, and a fixed administration time (10 min prior to the procedure). Administration of the study drug will be documented in the medical record and study case report forms, including dose, time of administration, and any deviations from protocol. Video and physiologic recordings of the procedure will be reviewed to confirm timing and completeness of administration. Any protocol deviations will be recorded and reviewed by the study team and reported to the DSMB as appropriate.

### Trial oversight and governance

This is a single-centre, investigator-initiated trial coordinated at IWK Health, which serves as the sole study site and coordinating centre. The Principal Investigator (HM) is responsible for overall trial conduct, protocol adherence, regulatory compliance, and reporting. Trial conduct will be monitored continuously by the Principal Investigator through review of recruitment, informed consent, intervention delivery, protocol deviations, and data completeness. Institutional research support services and the sponsor’s research office will perform periodic monitoring of source documentation, data entry, and storage procedures. Any issues identified will be documented and addressed through corrective actions as needed. Participant safety oversight is provided by the independent Data Safety Monitoring Board (DSMB) as described above, and the Research Ethics Board and regulatory authorities may audit the study at any time.

Any important protocol modifications (e.g., changes to eligibility criteria, interventions, outcomes, or safety procedures) will be submitted for approval to the IWK Health Research Ethics Board and relevant regulatory authorities prior to implementation. Approved amendments will be communicated to all investigators and study personnel through written updates and training as required. When applicable, parents or legally authorized representatives will be informed of changes that may affect their infant’s participation or risk. The trial registry (ClinicalTrials.gov) will be updated to reflect all substantive protocol changes.

There are no provisions for financial compensation for research-related injury beyond standard medical care provided by the institution.

### Equity, diversity, and inclusion (PRO-EDI considerations)

This trial includes a population defined by clinical vulnerability rather than social characteristics. Eligibility criteria are based on gestational age and clinical status, without exclusion based on sex, race, ethnicity, or socioeconomic status. Interpretation services are provided to support equitable access to consent processes for non-English-speaking families. Participant characteristics relevant to equity, including sex and gestational age, will be reported and considered in analyses. Recruitment and consent procedures have been designed to minimize barriers to participation and ensure inclusive engagement of families.

Patients and the public were not involved in the initial design of this study. However, parent partners will be involved in knowledge translation and dissemination of study findings. Specifically, parent partners will contribute to the development of plain-language summaries for families, advise on strategies for communicating results to participants, and support dissemination to the broader public and family communities. This involvement will ensure that study findings are accessible, relevant, and meaningful to families of preterm infants.

### Dissemination of results

The results of this trial will be disseminated through publication in a peer-reviewed journal and presentation at national and international scientific meetings. Study findings will be reported in the ClinicalTrials.gov registry in accordance with reporting requirements. A plain-language summary of the results will be developed and shared with participating families. Findings will also be communicated to healthcare professionals through institutional rounds, professional networks, and relevant clinical forums. Parent partners will be involved in knowledge translation activities to support dissemination to families and the broader public.

## Discussion

Procedural pain management remains a critical challenge in neonatal intensive care, particularly for preterm infants who undergo repeated invasive interventions during a vulnerable period of neurodevelopment. Evidence indicates that early and repeated exposure to procedural pain is associated with altered brain development, dysregulated stress responses, and adverse neurodevelopmental and behavioral outcomes later in life [1–6, 27]. Despite increased awareness and widespread use of non-pharmacologic comfort measures, effective analgesia for invasive procedures remains inconsistent.

Retinopathy of prematurity screening is a necessary routine procedure in preterm infants and is consistently associated with elevated pain scores, physiologic instability, and prolonged crying [10, 11, 13, 28]. Standard practice typically includes oral sucrose, non-nutritive sucking, swaddling, and topical anesthetic eye drops; however, these interventions alone have not reliably reduced pain to mild or no pain levels during ROP examinations [9, 16, 16, 29–32]. There is a need for effective and safe analgesic strategies that can be feasibly integrated into routine ROP screening.

Intranasal fentanyl represents a potential option for neonatal procedural analgesia due to its rapid onset of action, high bioavailability, and avoidance of intravenous access [18–20]. In pediatric emergency and peri-procedural settings, intranasal fentanyl has demonstrated efficacy and an acceptable safety profile [19, 21, 22]. However, evidence supporting its use in preterm infants remains limited. Two randomized controlled trials evaluating intranasal fentanyl during ROP screening have reported mixed findings, with uncertainty regarding both analgesic effectiveness and safety outcomes [13, 24]. In addition to methodological differences, these studies enrolled heterogeneous patient populations across different NICU settings, with variability in gestational age ranges, clinical stability, baseline pain management practices, and healthcare contexts, which may limit generalizability. Variations in pain assessment methods, dosing regimens, timing of administration, and safety monitoring further complicate interpretation of results, underscoring the need for additional high-quality, contextually rigorous trials in this vulnerable population.

The present trial is designed to address these gaps through randomization, double blinding, standardized dosing and timing, comprehensive pain assessment using the Premature Infant Pain Profile–Revised (PIPP-R) [33], and detailed safety monitoring. The use of synchronized video, audio, and physiologic monitoring enables blinded assessment of behavioral and physiologic indicators of pain throughout the procedure and recovery period, enhancing outcome validity. In addition, measurement of salivary cortisol provides an objective biological marker of stress response that complements behavioral pain scores and has been used in prior neonatal pain research^34^–^37^.

Safety considerations are central to this trial given the vulnerability of the study population and the use of an opioid analgesic. Conservative dosing, strict eligibility criteria, continuous physiologic monitoring, predefined adverse event definitions, and oversight by an independent Data Safety Monitoring Board have been incorporated to minimize risk [17, 20, 23, 38, 39]. Adjustment for prior painful exposures and exam number further strengthens the analytic approach by accounting for inter-infant variability in pain sensitivity and stress responsivity [3, 6].

This study also incorporates several practical considerations relevant to implementation within a busy neonatal intensive care environment. The intervention was designed to align with existing clinical workflows by using a rapid, non-invasive administration route that does not require intravenous access or substantial additional procedural time. Nonetheless, conducting procedural pain research in medically fragile preterm infants presents operational challenges, including coordination of time-sensitive study procedures with routine clinical care, maintaining blinding during medication preparation and administration, and ensuring high-quality synchronized physiologic and video data collection during bedside examinations. Recruitment may also be influenced by parental stress, clinical acuity, and the unpredictable timing of ROP screening procedures. These considerations were incorporated into protocol development through close collaboration with neonatal clinicians, pharmacy services, ophthalmology, and research personnel to support feasibility and protocol adherence within the NICU setting.

The findings of this trial have the potential to inform clinical practice and guideline development for pain management during ROP screening. Whether intranasal fentanyl demonstrates clinically meaningful pain reduction or reveals limitations in effectiveness or safety, the results will contribute important evidence to guide clinical decision-making and future research in neonatal procedural pain management.

## Trial status

This protocol is dated 15 May 2026. At the time of submission, participant recruitment has not yet begun. The study has received Health Canada authorization and institutional Research Ethics Board approval, and recruitment is anticipated to begin following completion of site start-up activities. Recruitment is expected to be completed by June 2027.

## Supplementary Information

Supplementary Material 1.

## Data Availability

No datasets were generated or analysed during the current study.

## Appendix

### Classification of prior painful procedures

These categories were predefined to standardize abstraction of cumulative procedural exposure in this preterm population. Prior procedures will be abstracted from the medical record and classified as tissue-breaking, invasive, or potentially noxious interventions, grouped according to clinical domain. Classification reflects potential exposure to procedural stress or nociceptive stimulation and does not imply equivalent pain intensity across categories.

#### Tissue-breaking vascular access

- Heel lance
- Arterial punctureddd
- Venous puncture
- Peripheral intravenous (IV) insertion or removal
- Long peripheral IV insertion or removal
- Peripheral arterial line insertion or removal
- Umbilical venous catheter (UVC) insertion or removal
- Umbilical arterial catheter (UAC) insertion or removal
- Peripherally inserted central catheter (PICC) insertion or removal
- Central venous line insertion or removal (internal jugular, subclavian, femoral)

#### Airway and respiratory procedures

- Endotracheal tube insertion or removal
- Tracheostomy insertion, change, or removal
- Laryngoscopy
- Surfactant administration
- LISA/MIST surfactant administration
- Suctioning

#### Surgical and drainage procedures

- Chest tube insertion or removal
- Pericardial catheter (pigtail) insertion or removal
- Jackson-Pratt (JP) drain insertion or removal
- Penrose drain insertion or removal
- Dialysis catheter insertion or removal
- Other surgical intervention (specify):

#### Gastrointestinal procedures

- Orogastric tube insertion
- Nasogastric tube insertion
- Nasojejunal tube insertion
- Gastrostomy tube insertion

#### Genitourinary procedures

- Urinary catheter insertion
- Suprapubic catheter insertion

#### Neurologic and ophthalmologic procedures

- Lumbar puncture
- Retinopathy of prematurity (ROP) examination
- Ventricular access device tap

#### Additional painful procedures

- Adhesive or tape removal
- Other clinically documented stressful procedures (specify)

#### Operational notes

- Only procedures occurring prior to the study ROP examination will be recorded.
- Each occurrence will be counted separately when documented.GG
- Classification is based on clinical documentation; no retrospective pain scoring will be assigned.

## Abbreviations

AE: Adverse event
BP: Blood pressure
CI: Confidence interval
DSMB: Data Safety Monitoring Board
GA: Gestational age
HR: Heart rate
IN: Intranasal
IWK: Isaac Walton Killam Health
MCG: micrograms
NICU: Neonatal Intensive Care Unit
NNS: Non—nutritive sucking
PIPP: R—Premature Infant Pain Profile—Revised
REB: Research Ethics Board
ROP: Retinopathy of prematurity
RR: Respiratory rate
SAE: Serious adverse event
SD: Standard deviation
SpO₂: Peripheral oxygen saturation
